# Supplementary material for: CTLA-4 antibody-drug conjugate reveals autologous destruction of B-lymphocytes associated with regulatory T cell impairment
Source: bioRxiv. 2023 Aug 29:2023.03.01.530608. Originally published 2023 Mar 2. Preprint. [Version 2] doi: 10.1101/2023.03.01.530608 (PMC10002750; doi:10.1101/2023.03.01.530608)
Supplement: 1 [file NIHPP2023.03.01.530608V2-supplement-1.pdf]

bioRxiv preprint doi: https://doi.org/10.1101/2023.03.01.530608; this version posted August 29, 2023. The copyright holder for this preprint (which was not certified by peer review) is the author/funder, who has granted bioRxiv a license to display the preprint in perpetuity. It is made available under aCC-BY 4.0 International license.

Table 1 Drug to antibody ratio (DAR)

Entry	A_{280}	A_{252}	CmAb (Moles)	CDM1(Moles)	DAR CDM1/CmAB
hIgGFC-DM1	0.51	0.48	6.92705E-06	1.1531E-05	1.7
Ipilimumab-DM1	0.62	0.42	2.30582E-06	7.32032E-06	3.2

Drug to antibody ratio (DAR)= CDM1/ CmAB. DM1 known extinction coefficients (A_{280}, 5700 $M^{-1}cm^{-1}$; A_{252}, 28084 $M^{-1}cm^{-1}$). Experimentally derived extinction coefficients for Ipilimumab (A_{280}, 242115 $M^{-1}cm^{-1}$; A_{252}, 92989 $M^{-1}cm^{-1}$) and hIgGFc (A_{280}, 64136 $M^{-1}cm^{-1}$; A_{252}, 22544 $M^{-1}cm^{-1}$)

Table S1. Drug to antibody ratio (DAR)

bioRxiv preprint doi: https://doi.org/10.1101/2023.03.01.530608; this version posted August 29, 2023. The copyright holder for this preprint (which was not certified by peer review) is the author/funder, who has granted bioRxiv a license to display the preprint in perpetuity. It is made available under aCC-BY 4.0 International license.

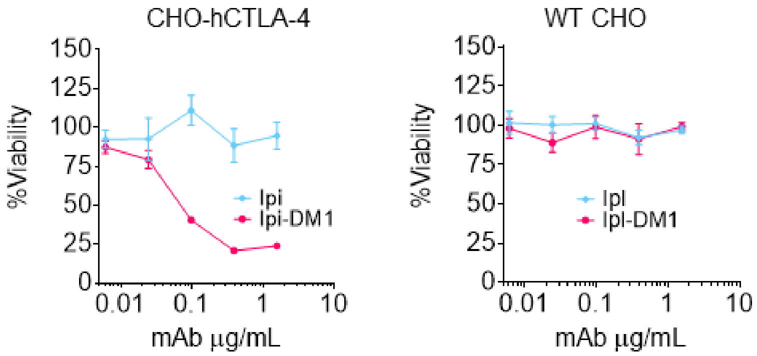

Figure 1-figure supplement 1. CTLA-4 antibody-drug conjugate killing is CTLA-4 specific. Cell viability of CHO-hCTLA-4 cells and wild type CHO cells after 72 hours incubation with Ipi or Ipi-DM1 as measured by MTT assay (n=2). Data representative of two or more repeats.

bioRxiv preprint doi: https://doi.org/10.1101/2023.03.01.530608; this version posted August 29, 2023. The copyright holder for this preprint (which was not certified by peer review) is the author/funder, who has granted bioRxiv a license to display the preprint in perpetuity. It is made available under aCC-BY 4.0 International license.

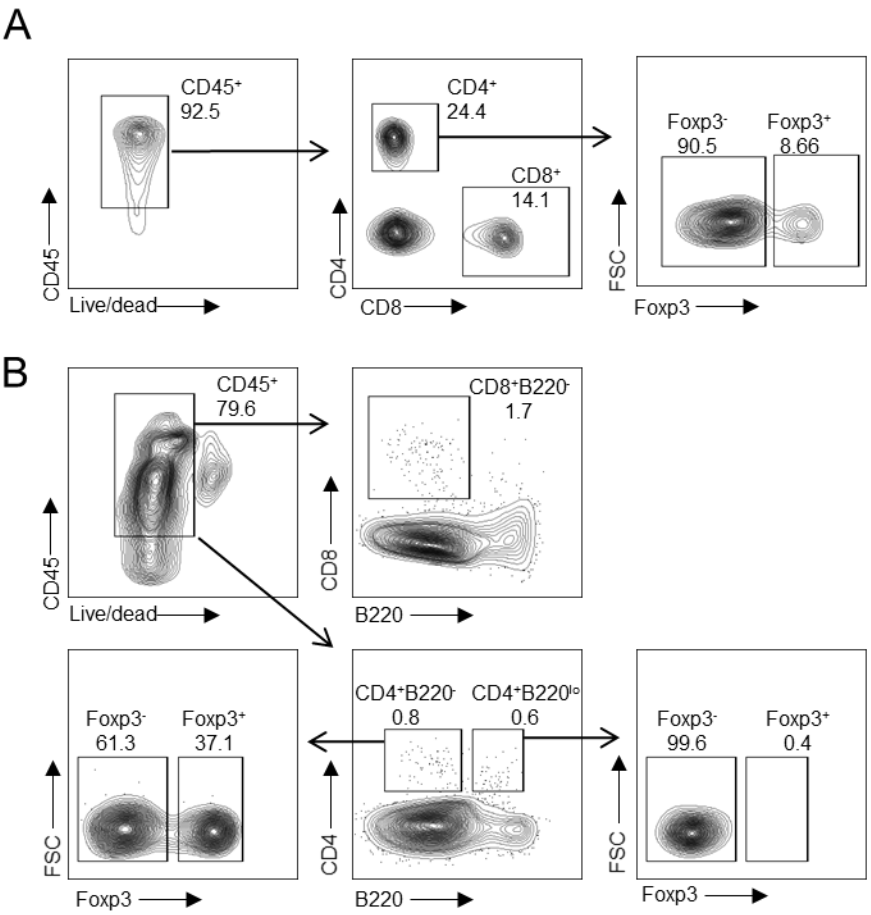

Figure 1-figure supplement 2. Gating strategy for T cells.

(A) Tregs ($CD4^+$ $Foxp3^+$) and CD4-nonTreg ($CD4^+$ $Foxp3^-$) in blood. (B) CD8-T ($CD4^+B220^-$), CD4-T ($CD4^+B220^-$), Tregs ($CD4^+B220^-Foxp3^+$) and CD4-nonTreg ($CD4^+B220^-Foxp3^-$) in bone marrow.

bioRxiv preprint doi: https://doi.org/10.1101/2023.03.01.530608; this version posted August 29, 2023. The copyright holder for this preprint (which was not certified by peer review) is the author/funder, who has granted bioRxiv a license to display the preprint in perpetuity. It is made available under aCC-BY 4.0 International license.

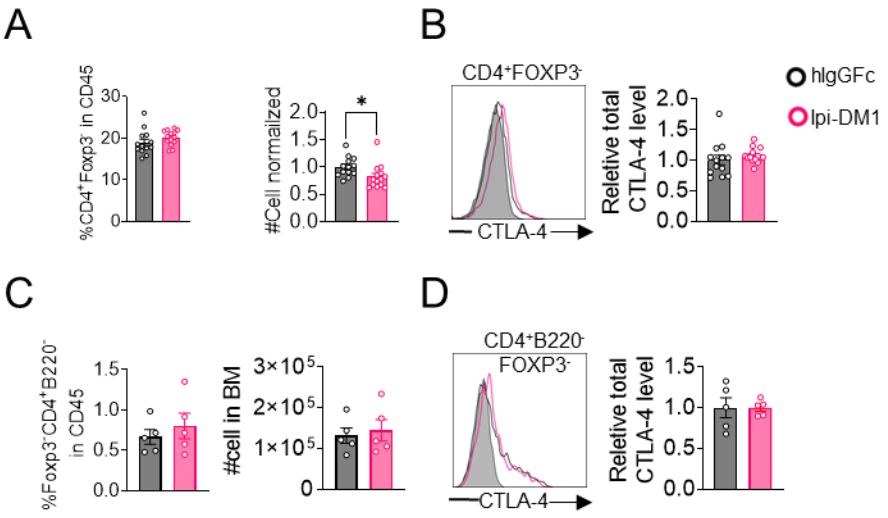

Figure 1-figure supplement 3. CD4-nonTregs and CTLA-4 expression.

(A, B) Blood, (A) % CD4$^+$ Foxp3$^-$ in CD45 and normalized cell number, (B) relative CTLA-4 level in CD4-nonTregs. (C, D) Bone marrow, (C) % CD4$^+$B220$^-$Foxp3$^-$ in CD45 and absolute cell number, (B) relative CTLA-4 level in CD4-nonTregs (CD4$^+$B220$^-$ Foxp3$^-$). (A, B) Data combined from two independent experiments (n=13-14). (C, D) Data representative of two independent experiments (n=5). Data analyzed using an unpaired two-tailed Student's t test and represented as mean ± SEM. *p < 0.05, **p < 0.01, ***p < 0.001, ****p < 0.0001.

bioRxiv preprint doi: https://doi.org/10.1101/2023.03.01.530608; this version posted August 29, 2023. The copyright holder for this preprint (which was not certified by peer review) is the author/funder, who has granted bioRxiv a license to display the preprint in perpetuity. It is made available under aCC-BY 4.0 International license.

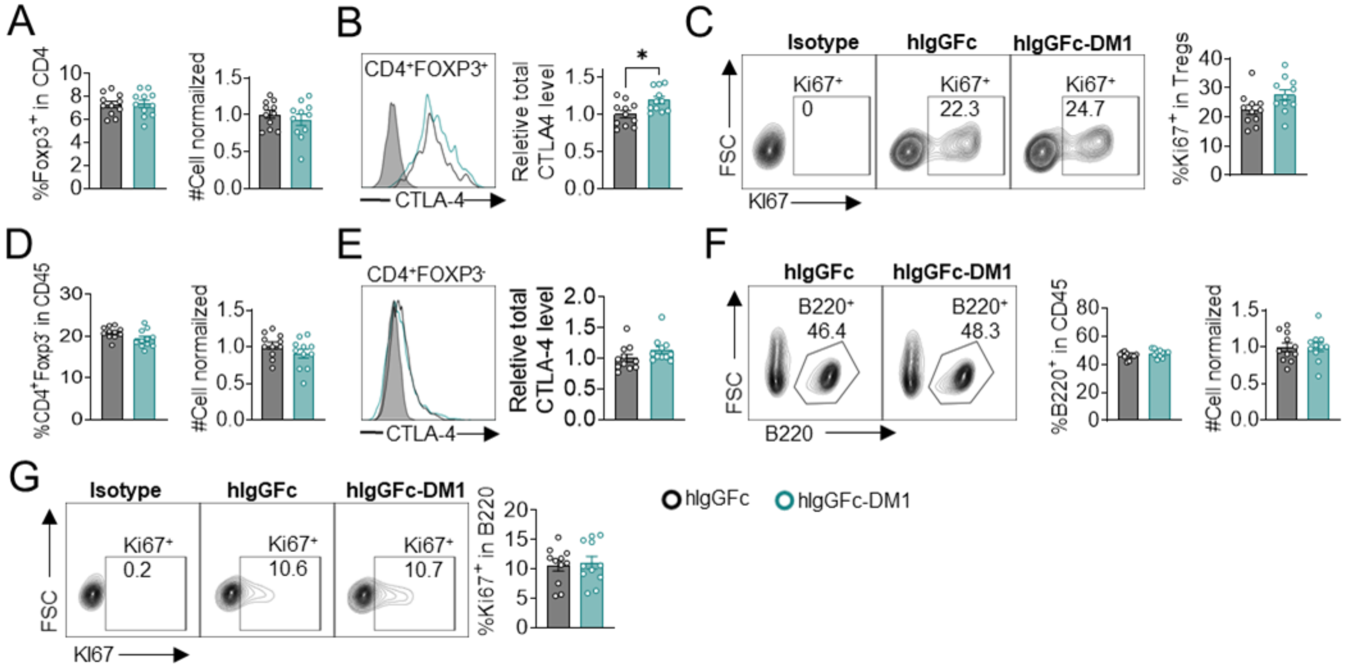

Figure 1-figure supplement 4. B-cell depletion is not mediated by DM1 payload off-target release.

Mice treated with hIgGFc or hIgGFc-DM1, blood flow analysis on day 9. (A) % Foxp3$^+$ in CD4 and normalized cell number. (B) Relative CTLA-4 level in Tregs. (C) % Ki67$^+$ in Tregs. (D) % CD4$^+$ Foxp3$^-$ in CD45 and normalized cell number. (E) Relative CTLA-4 level in CD4-nonTregs. (F) FACS profiles depicting gating strategy after gating on CD45$^+$ and data summaries of % B220$^+$ in CD45 and normalized cell number. (G) % Ki67$^+$ in B cells. Data combined from two independent experiments (n=11) and analyzed using an unpaired two-tailed Student's t test and represented as mean ± SEM. *$p < 0.05$, **$p < 0.01$, ***$p < 0.001$, ****$p < 0.0001$.

bioRxiv preprint doi: https://doi.org/10.1101/2023.03.01.530608; this version posted August 29, 2023. The copyright holder for this preprint (which was not certified by peer review) is the author/funder, who has granted bioRxiv a license to display the preprint in perpetuity. It is made available under aCC-BY 4.0 International license.

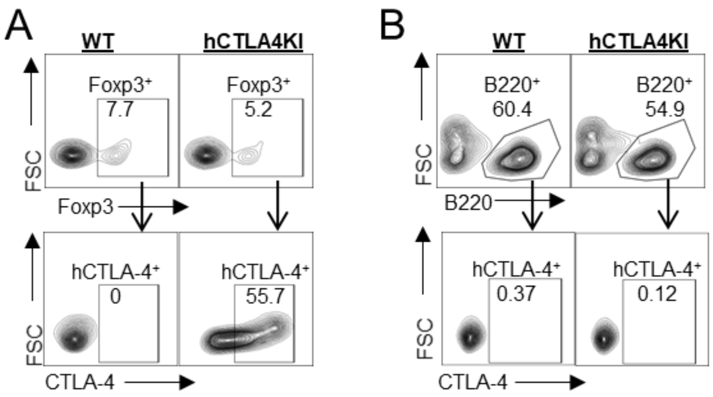

Figure 1-figure supplement 5. B-cells do not express CTLA-4.

FACS profile of human CTLA-4 in wild-type and human CTLA-4 knock-in mice peripheral blood, (A) % CTLA-4^+ in Tregs ($CD45^+$ $CD4^+$ $Foxp3^+$), and (B) % CTLA-4^+ in B cells ($CD45^+$ $B220^+$). Data representative of 2 mice.

bioRxiv preprint doi: https://doi.org/10.1101/2023.03.01.530608; this version posted August 29, 2023. The copyright holder for this preprint (which was not certified by peer review) is the author/funder, who has granted bioRxiv a license to display the preprint in perpetuity. It is made available under aCC-BY 4.0 International license.

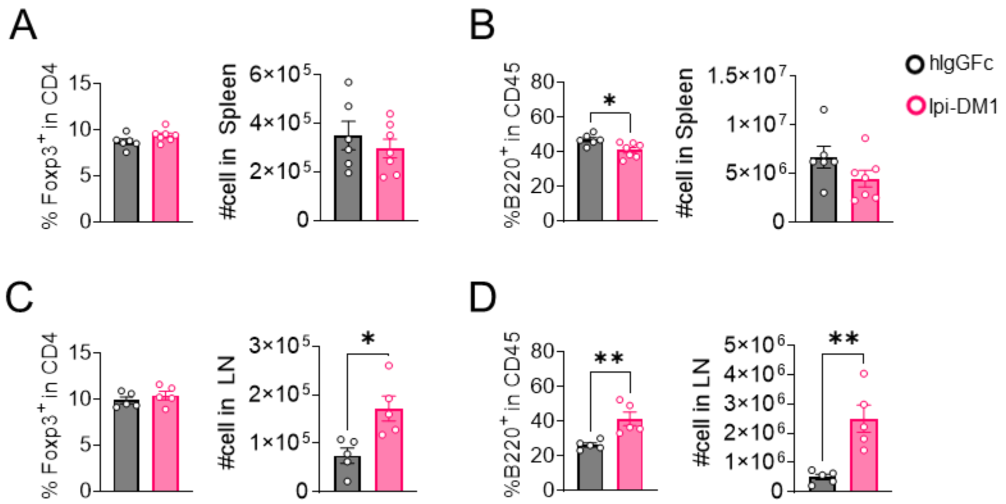

Figure 1-figure supplement 6. Tregs and B cells in spleen and lymph nodes.

Analysis of spleen and lymph nodes from *Ctla4*$^{h/h}$ mice on day 9 after treatment with hIgGFc or Ipi-DM1 ADC. (A, B) Spleen, (A) % Foxp3+ in CD4 and absolute cell number, (B) % B220+ in CD45 and absolute cell number. (C, D) combined inguinal and cervical lymph nodes, (C) % Foxp3+ in CD4 and absolute cell number, (D) % B220+ in CD45 and absolute cell number. Data representative of two independent experiments (n=5-7) and analyzed using an unpaired two-tailed Student's t test and represented as mean ± SEM. Non-significant [ns], *p < 0.05, **p < 0.01, ***p < 0.001, ****p < 0.0001.

bioRxiv preprint doi: https://doi.org/10.1101/2023.03.01.530608; this version posted August 29, 2023. The copyright holder for this preprint (which was not certified by peer review) is the author/funder, who has granted bioRxiv a license to display the preprint in perpetuity. It is made available under aCC-BY 4.0 International license.

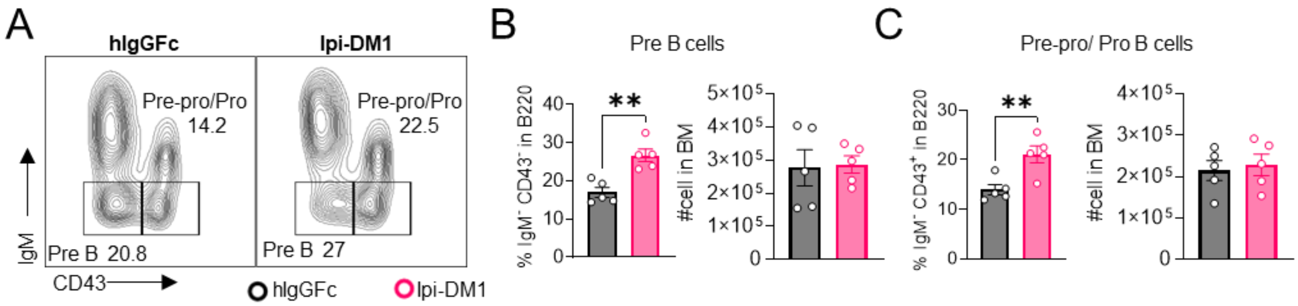

Figure 1-figure supplement 7. IgM negative B-cell subtypes in bone marrow.

(A) FACS profile gating of Pre B (IgM^-CD43^-) and Pre-pro/Pro (IgM^-CD43^+) cells in B220. (B-C) % of B cell subtype in B220 and absolute cell numbers, (B) Pre B cells, (C) Pre-pro/Pro B cells. Data representative of two independent experiments (n=5) and analyzed using an unpaired two-tailed Student's t test and represented as mean ± SEM. *$p < 0.05$, **$p < 0.01$, ***$p < 0.001$, ****$p < 0.0001$.

bioRxiv preprint doi: https://doi.org/10.1101/2023.03.01.530608; this version posted August 29, 2023. The copyright holder for this preprint (which was not certified by peer review) is the author/funder, who has granted bioRxiv a license to display the preprint in perpetuity. It is made available under aCC-BY 4.0 International license.

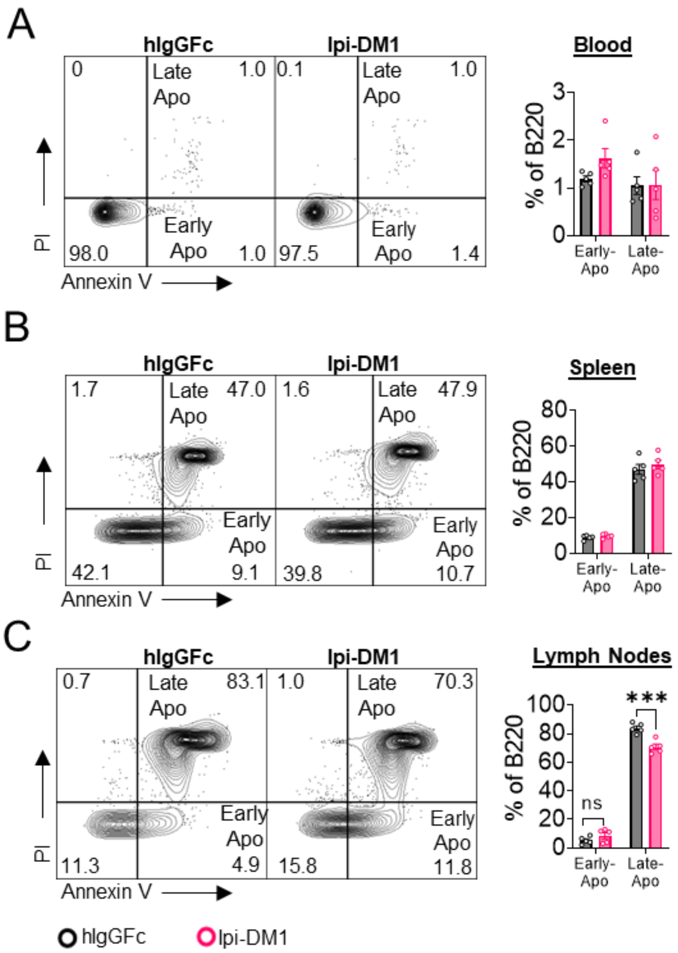

Figure 2-figure supplement 1. B cell apoptosis in blood and lymphoid organs.

B cell apoptosis on day 9 after treatment with hIgGFc or Ipi-DM1 ADC. (A) B220^{+} blood cells. (B) B220^{+} spleen cells. (C) B220^{+} lymph node cells. Data representative of two independent experiments (n=5) and analyzed using an unpaired two-tailed Student's t test and represented as mean ± SEM. Non-significant [ns], *p < 0.05, **p < 0.01, ***p < 0.001, ****p < 0.0001.

bioRxiv preprint doi: https://doi.org/10.1101/2023.03.01.530608; this version posted August 29, 2023. The copyright holder for this preprint (which was not certified by peer review) is the author/funder, who has granted bioRxiv a license to display the preprint in perpetuity. It is made available under aCC-BY 4.0 International license.

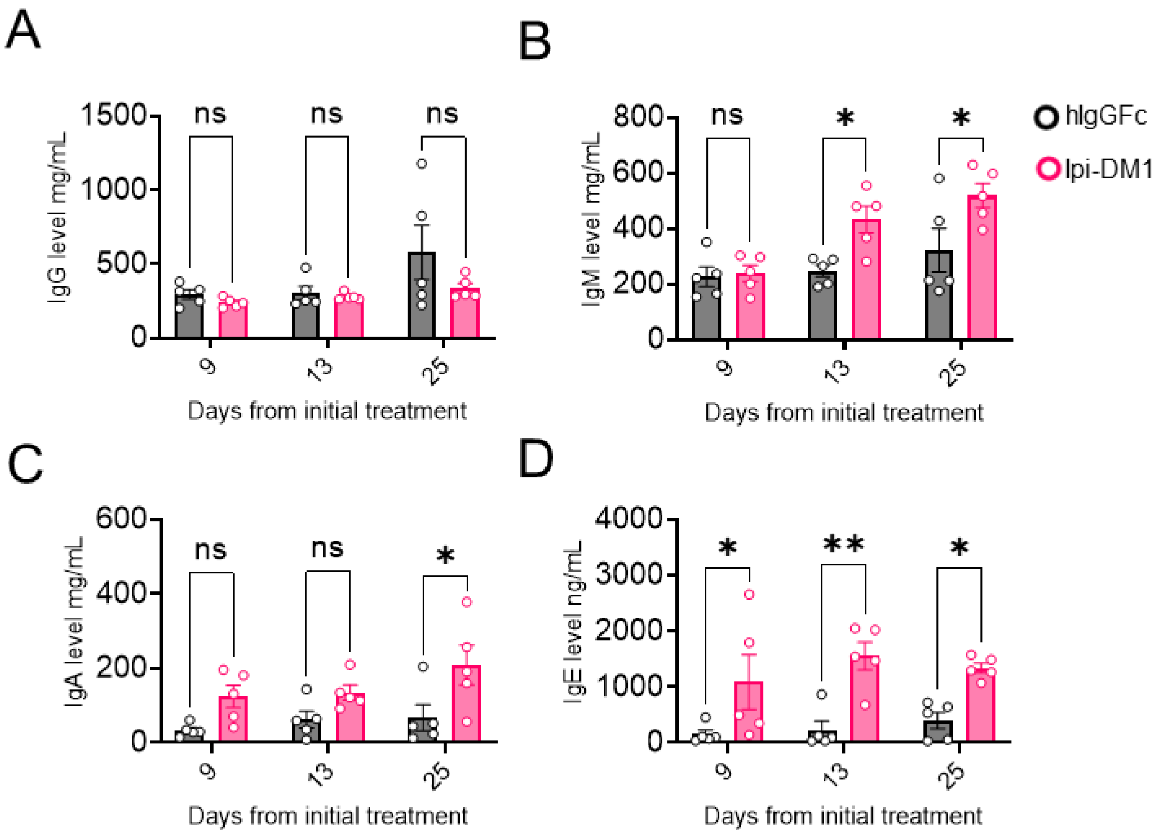

Figure 3-figure supplement 1. Treg impairment increases plasma immunoglobulins.

Mice treatment with hIgGFc or Ipi-DM1 ADC and plasma collected on day 9, 13, 25 for ELISA Ig level quantification. (A) IgG. (B) IgM. (C) IgA. (D) IgE. Data representative of two independent experiments (n=5) and analyzed using ordinary two-way Anova with Bonferroni's multiple comparisons test and as mean ± SEM. Non-significant [ns], *p < 0.0332, **p < 0.0021, ***p < 0.0002, ****p < 0.0001.

bioRxiv preprint doi: https://doi.org/10.1101/2023.03.01.530608; this version posted August 29, 2023. The copyright holder for this preprint (which was not certified by peer review) is the author/funder, who has granted bioRxiv a license to display the preprint in perpetuity. It is made available under aCC-BY 4.0 International license.

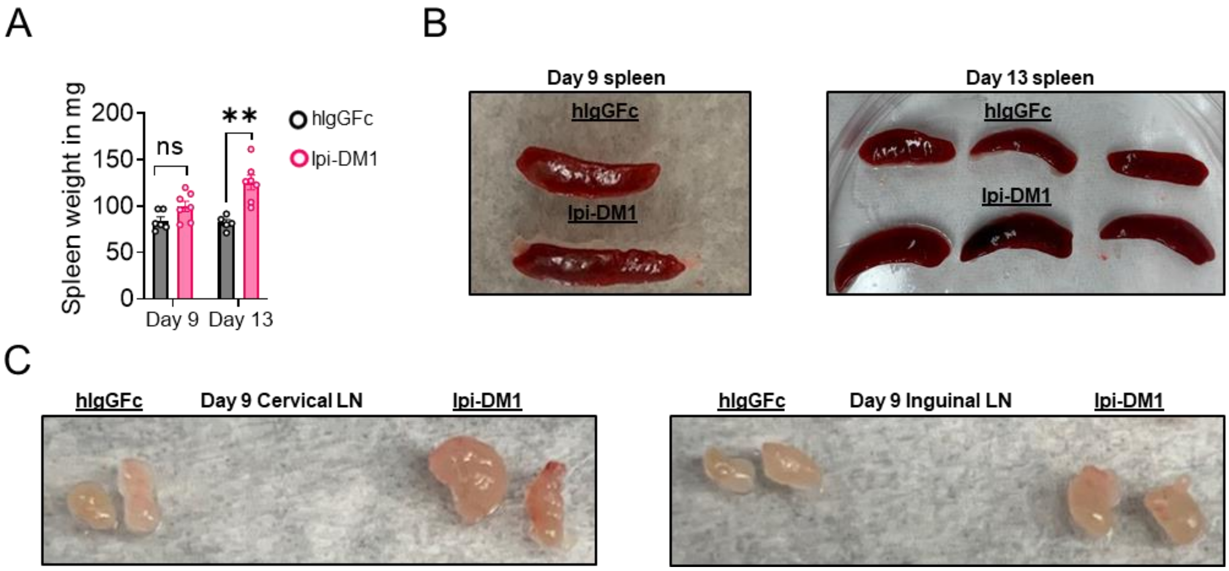

Figure 3-figure supplement 2. CTLA-4 antibody-drug conjugate leads to enlarged spleen and lymph nodes.

Day 9 or 13 after treatment with hIgGFc or Ipi-DM1 ADC. (A) Spleen weight. (B) Splenomegaly. (C) Lymphadenopathy. (A) Data representative of two independent experiments (n=5-7) and analyzed using an unpaired two-tailed Student's t test and represented as mean ± SEM. Non-significant [ns], *p < 0.05, **p < 0.01, ***p < 0.001, ****p < 0.0001.

bioRxiv preprint doi: https://doi.org/10.1101/2023.03.01.530608; this version posted August 29, 2023. The copyright holder for this preprint (which was not certified by peer review) is the author/funder, who has granted bioRxiv a license to display the preprint in perpetuity. It is made available under aCC-BY 4.0 International license.

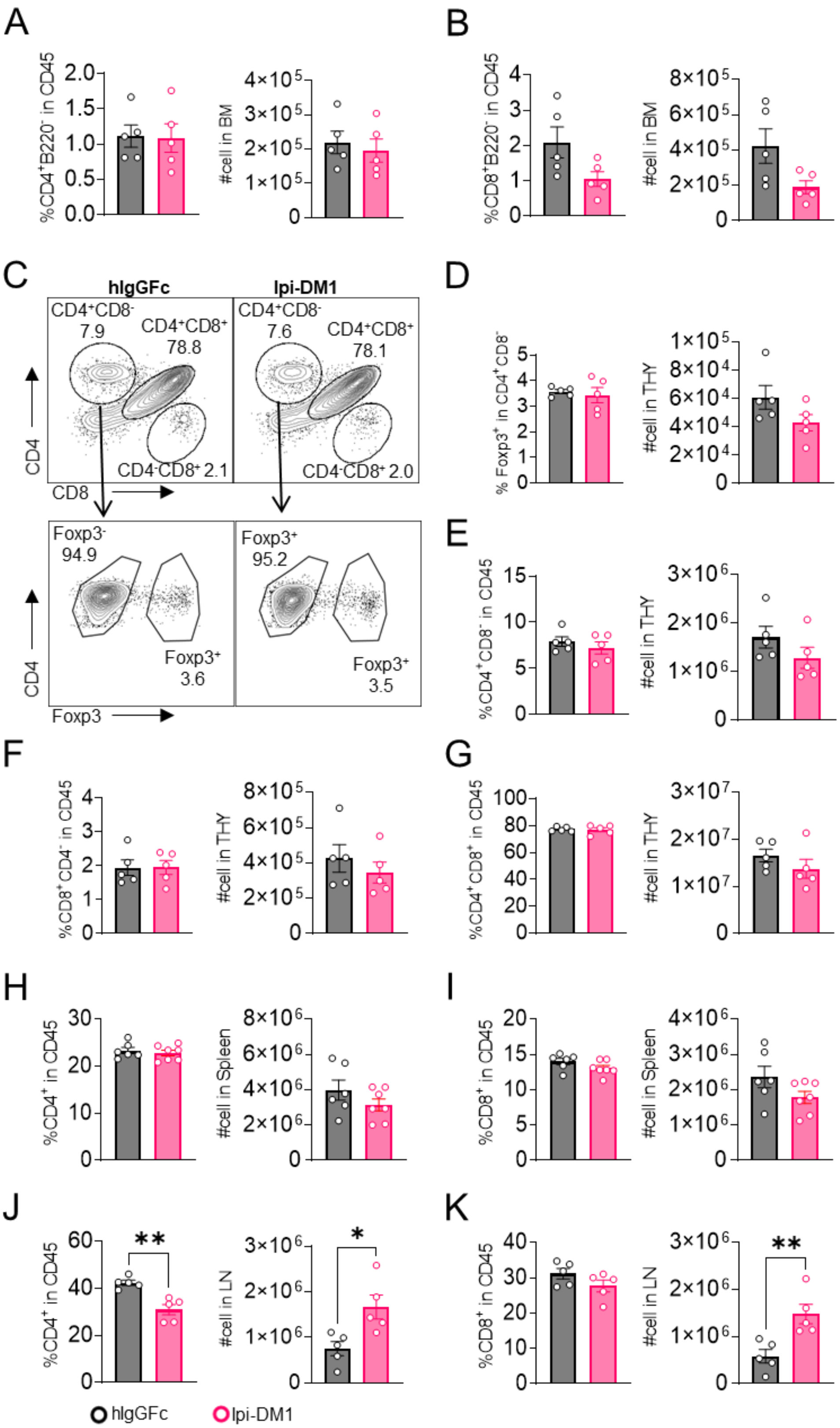

799

800

bioRxiv preprint doi: https://doi.org/10.1101/2023.03.01.530608; this version posted August 29, 2023. The copyright holder for this preprint (which was not certified by peer review) is the author/funder, who has granted bioRxiv a license to display the preprint in perpetuity. It is made available under aCC-BY 4.0 International license.

Figure 4-figure supplement 1. CTLA-4 antibody-drug conjugate impact on T cells in lymphoid organs.

Flow analysis of T cells at day 9 after treatment with hIgGFc or Ipi-DM1 ADC. (A, B) Bone marrow, (C-G) thymus, (H, I) spleen, (J, K) combined inguinal and cervical lymph nodes. (A) % CD4^{+}B220^{-} in CD45 and absolute cell number. (B) % CD8^{+}B220^{-} in CD45 and absolute cell number. (C) Gating strategy defining T-cell types in CD45^{+} cells in thymus. (D) Tregs (% Foxp3^{+} in CD4^{+}CD8^{-}) and absolute cell number. (E) % CD4^{+}CD8^{-} in CD45 and absolute cell number. (F) % CD8^{+}CD4^{-} in CD45 and absolute cell number. (G) % CD4^{+}CD8^{+} in CD45 and absolute cell number. (H, J) % CD4^{+} in CD45 and absolute cell number. (I, K) % CD8^{+} in CD45 and absolute cell number. Data representative of two independent experiments (n=5-7) and analyzed using an unpaired two-tailed Student's t test and represented as mean ± SEM. *$p < 0.05$, **$p < 0.01$, ***$p < 0.001$, ****$p < 0.0001$.

bioRxiv preprint doi: https://doi.org/10.1101/2023.03.01.530608; this version posted August 29, 2023. The copyright holder for this preprint (which was not certified by peer review) is the author/funder, who has granted bioRxiv a license to display the preprint in perpetuity. It is made available under aCC-BY 4.0 International license.

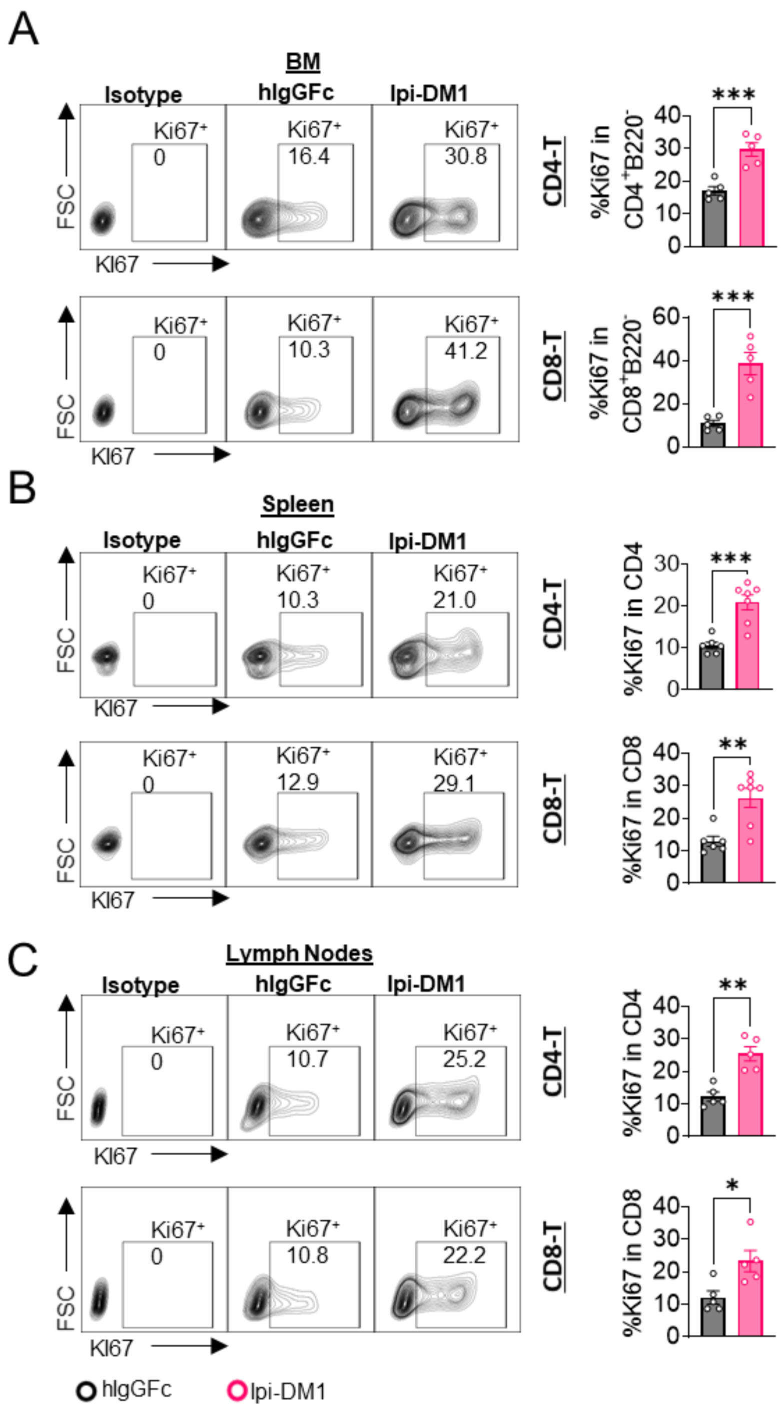

813

814 **Figure 4-figure supplement 2. CTLA-4 antibody-drug conjugate increase T cell**

815 **proliferation in lymphoid organs.**

bioRxiv preprint doi: https://doi.org/10.1101/2023.03.01.530608; this version posted August 29, 2023. The copyright holder for this preprint (which was not certified by peer review) is the author/funder, who has granted bioRxiv a license to display the preprint in perpetuity. It is made available under aCC-BY 4.0 International license.

Flow analysis at day 9 after treatment with hIgGFc or Ipi-DM1 ADC. (A) % $Ki67^+$ in $CD4^+B220^-$ and $CD8^+B220^-$ T cells in bone marrow. (B) % $Ki67^+$ in CD4 and CD8 T cells in spleen. (C) % $Ki67^+$ in CD4 and CD8 T cells in lymph nodes. Data representative of two independent experiments (n=5-7) and analyzed using an unpaired two-tailed Student's t test and represented as mean ± SEM. *$p < 0.05$, **$p < 0.01$, ***$p < 0.001$, ****$p < 0.0001$.

bioRxiv preprint doi: https://doi.org/10.1101/2023.03.01.530608; this version posted August 29, 2023. The copyright holder for this preprint (which was not certified by peer review) is the author/funder, who has granted bioRxiv a license to display the preprint in perpetuity. It is made available under aCC-BY 4.0 International license.

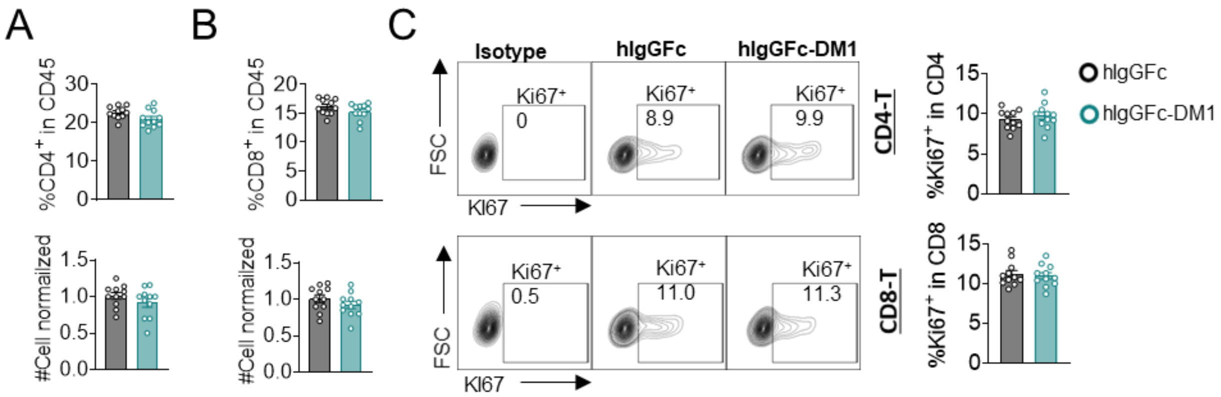

Figure 4-figure supplement 3. T-cell quantity and proliferation are not impacted by IgG-DM1 treatment.

Mice treated with hIgGFc or hIgGFc-DM1, blood flow analysis on day 9. (A) % CD4$^+$ in CD45 and normalized cell number. (B) % CD8$^+$ in CD45 and normalized cell number. (C) % Ki67$^+$ in CD4 and CD8 T cells. Data combined from two independent experiments (n=11) and analyzed using an unpaired two-tailed Student's t test and represented as mean ± SEM. *p < 0.05, **p < 0.01, ***p < 0.001, ****p < 0.0001.

bioRxiv preprint doi: https://doi.org/10.1101/2023.03.01.530608; this version posted August 29, 2023. The copyright holder for this preprint (which was not certified by peer review) is the author/funder, who has granted bioRxiv a license to display the preprint in perpetuity. It is made available under aCC-BY 4.0 International license.

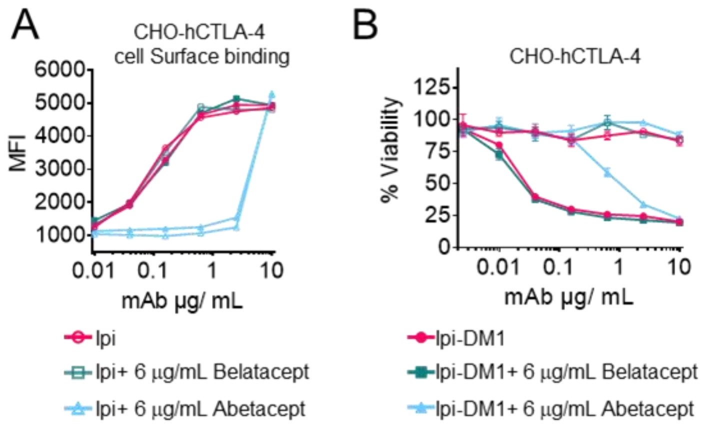

Figure 6-figure supplement 1. Mutant soluble CTLA-4-Ig does not neutralize Ipilimumab or its drug-conjugate.

(A) Detached CHO cells expressing hCTLA-4 were incubated with serial dilution of Ipi or Ipi-DM1 in the presence of given dose of Abatacept or Belatacept on ice for 30 minutes followed by FACS detection using mouse anti-human IgG AF488, mean fluorescence intensity (MFI) . (B) MTT cell viability of CHO-hCTLA-4 cells after 72 hours incubation with Ipi or Ipi-DM1 in the presence of given dose of Abatacept or Belatacept. Data representative of two independent experiments (n=3).

bioRxiv preprint doi: https://doi.org/10.1101/2023.03.01.530608; this version posted August 29, 2023. The copyright holder for this preprint (which was not certified by peer review) is the author/funder, who has granted bioRxiv a license to display the preprint in perpetuity. It is made available under aCC-BY 4.0 International license.

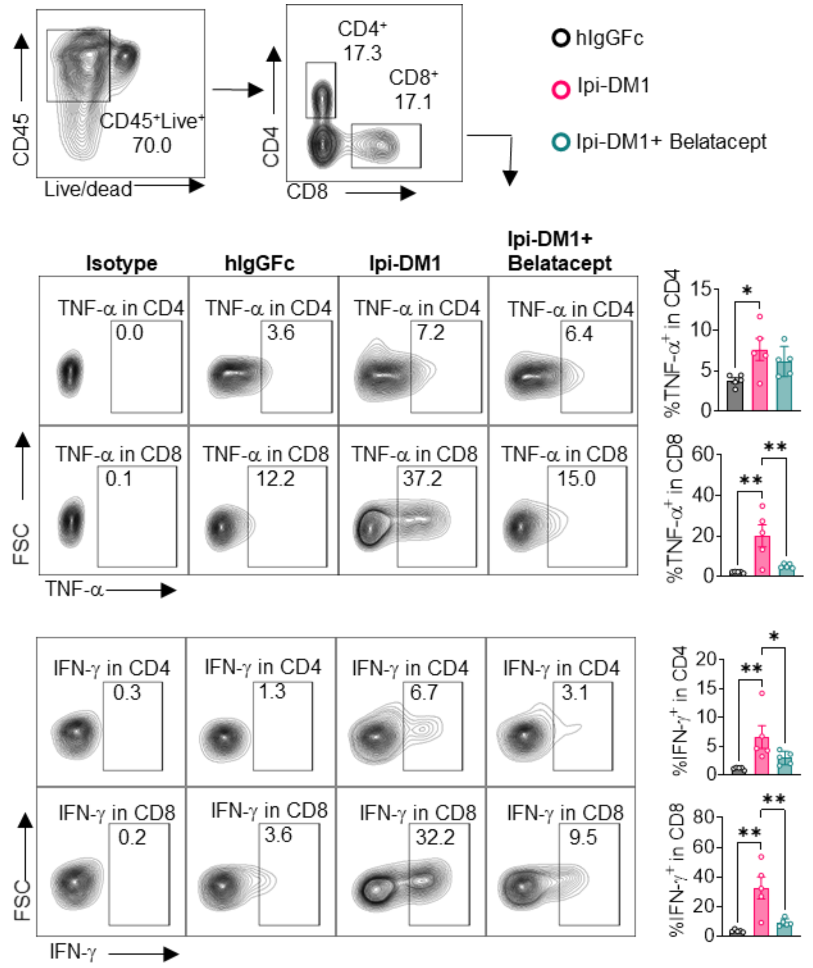

Figure 6-figure supplement 2. T-cell cytokine production.

Day 13 peripheral blood samples from treated (hIgGFc or Ipi-DM1 with/out Belatacept) mice post ACK buffer red blood lysis were cultured and stimulated in the presence of Iononmycin/PMA, and GolgiPlug for 4 hrs followed by intracellular cytokine detection (TNF-alpha, IFN-gamma) in CD4 and CD8 T cells. Data is representative of two independent experiments (n=5) analyzed by ordinary one-way ANOVA with Tukey's multiple comparisons test and represented as mean ± SEM. *$p < 0.05$, **$p < 0.01$, ***$p < 0.001$, ****$p < 0.0001$.

bioRxiv preprint doi: https://doi.org/10.1101/2023.03.01.530608; this version posted August 29, 2023. The copyright holder for this preprint (which was not certified by peer review) is the author/funder, who has granted bioRxiv a license to display the preprint in perpetuity. It is made available under aCC-BY 4.0 International license.

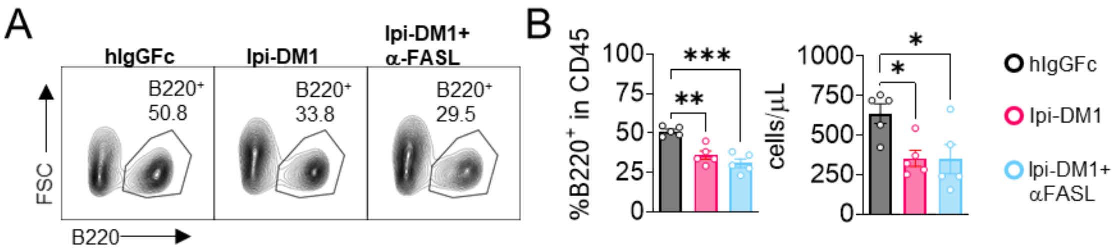

Figure 7-figure supplement 1. Anti-FASL fails to rescue B cell.

Ctla4$^{h/h}$ mice were treated intraperitoneally (i.p.) (100 µg/mouse) with hIgGFc or Ipi-DM1 with/out (100 µg/mouse of anti-FASL) every three days for a total of three doses and mice were bled on day 9. (A) FACS profile after gating on CD45 for B cells. (B) % B220^{+} in CD45 and absolute B cell number summaries. Data representative of one experiment (n=5) analyzed by ordinary one-way ANOVA with Tukey's multiple comparisons test and represented as mean ± SEM. *p < 0.05, **p < 0.01, ***p < 0.001, ****p < 0.0001.
