# Supplementary material for: CTLA-4 antibody-drug conjugate reveals autologous destruction of B-lymphocytes associated with regulatory T cell impairment
Source: bioRxiv. 2023 Mar 2:2023.03.01.530608. Preprint. [Version 1] doi: 10.1101/2023.03.01.530608 (PMC10002750; doi:10.1101/2023.03.01.530608)
Supplement: Supplementary file 1 [file NIHPP2023.03.01.530608v1-supplement-1.pdf]

bioRxiv preprint doi: https://doi.org/10.1101/2023.03.01.530608; this version posted March 2, 2023. The copyright holder for this preprint (which was not certified by peer review) is the author/funder, who has granted bioRxiv a license to display the preprint in perpetuity. It is made available under aCC-BY 4.0 International license.

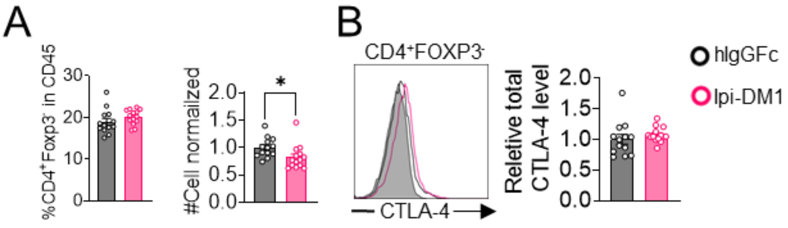

Figure 1-figure supplement 1. CD4-nonTregs and CTLA-4 expression.

(A) % $CD4^{+}$ $Foxp3^{-}$ in CD45 and normalized cell number. (B) Relative CTLA-4 level in CD4-nonTregs. Data combined from two independent experiments (n=13-14) and analyzed using an unpaired two-tailed Student's t test and represented as mean ± SEM. *p < 0.05, **p < 0.01, ***p < 0.001, ****p < 0.0001.

bioRxiv preprint doi: https://doi.org/10.1101/2023.03.01.530608; this version posted March 2, 2023. The copyright holder for this preprint (which was not certified by peer review) is the author/funder, who has granted bioRxiv a license to display the preprint in perpetuity. It is made available under aCC-BY 4.0 International license.

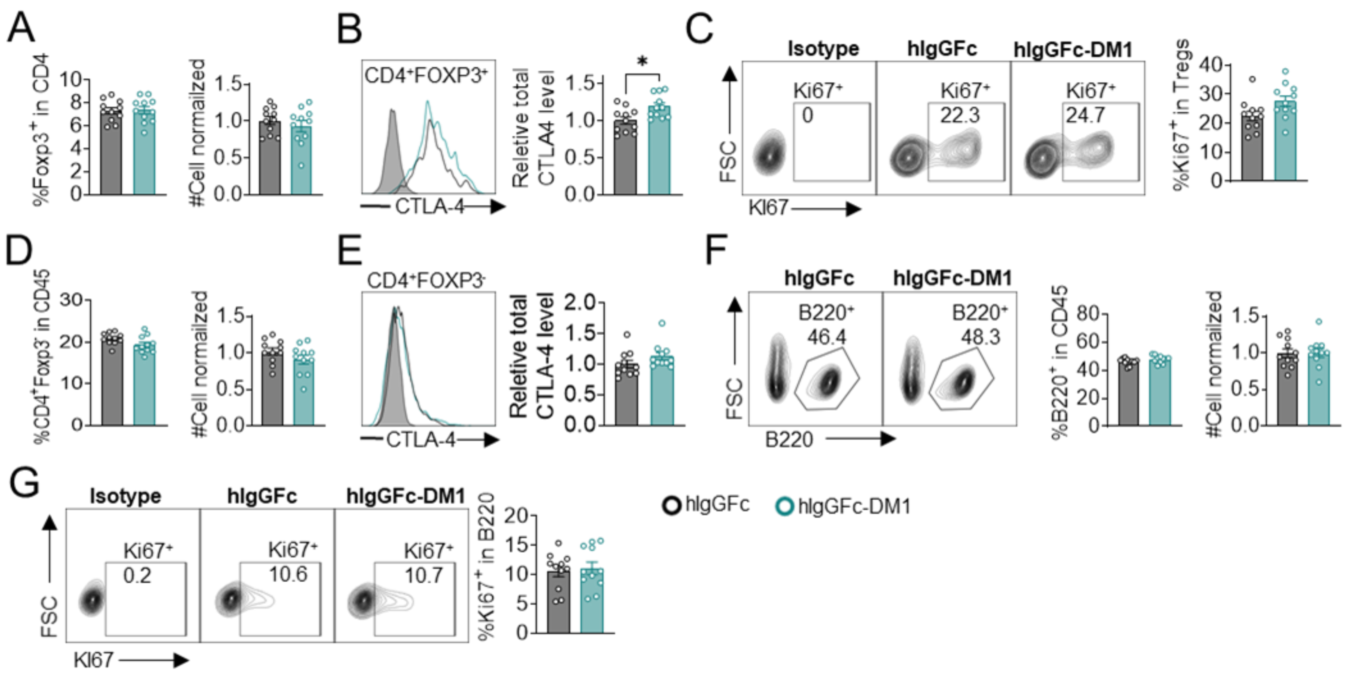

Figure 1-figure supplement 2. B-cell depletion is not mediated by DM1 payload off-target release.

(A) % Foxp3^{+} in CD4 and normalized cell number. (B) Relative CTLA-4 level in Tregs. (C) % Ki67^{+} in Tregs. (D) % CD4^{+} Foxp3^{-} in CD45 and normalized cell number. (E) Relative CTLA-4 level in CD4-nonTregs. (F) FACS profiles depicting gating strategy after gating on CD45^{+} and data summaries of % B220^{+} in CD45 and normalized cell number. (G) % Ki67^{+} in B cells. Data combined from two independent (n=11) and analyzed using an unpaired two-tailed Student's t test and represented as mean ± SEM. *p < 0.05, **p < 0.01, ***p < 0.001, ****p < 0.0001.

bioRxiv preprint doi: https://doi.org/10.1101/2023.03.01.530608; this version posted March 2, 2023. The copyright holder for this preprint (which was not certified by peer review) is the author/funder, who has granted bioRxiv a license to display the preprint in perpetuity. It is made available under aCC-BY 4.0 International license.

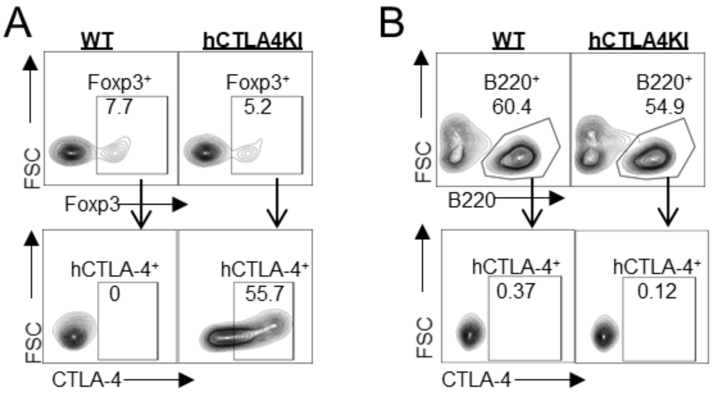

Figure 1-figure supplement 3. B-cells do not express CTLA-4.

FACS profile of human CTLA-4 in wild-type and human CTLA-4 knock-in mice peripheral blood, (A) % CTLA-4^{+} in Tregs (CD45^{+} CD4^{+} Foxp3^{+}), and (B) % CTLA-4^{+} in B cells (CD45^{+} B220^{+}). Data representative of 2 mice.

bioRxiv preprint doi: https://doi.org/10.1101/2023.03.01.530608; this version posted March 2, 2023. The copyright holder for this preprint (which was not certified by peer review) is the author/funder, who has granted bioRxiv a license to display the preprint in perpetuity. It is made available under aCC-BY 4.0 International license.

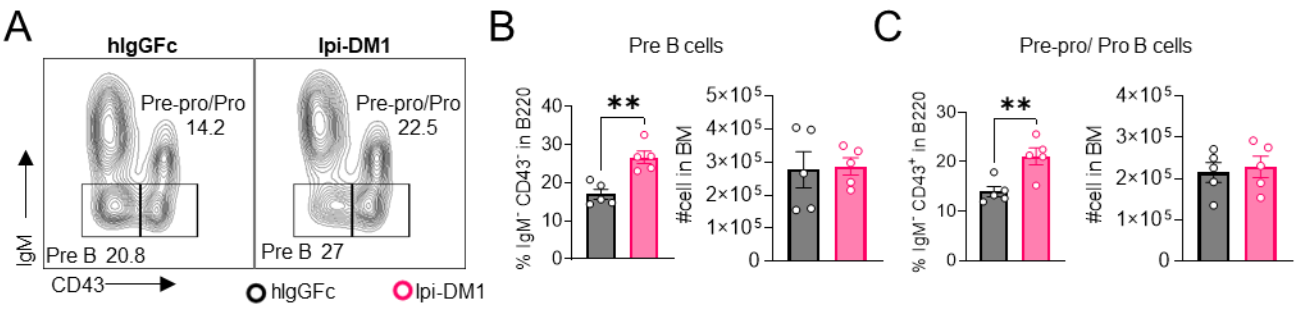

Figure 1-figure supplement 4. IgM negative B-cell subtypes in bone marrow.

(A) FACS profile gating of Pre B (IgM^-CD43^-) and Pre-pro/Pro (IgM^-CD43^+) cells in B220. (B-C) % of B cell subtype in B220 and absolute cell numbers, (B) Pre B cells, (C) Pre-pro/Pro B cells. Data is representative of one experiment (n=5) and analyzed using an unpaired two-tailed Student's t test and represented as mean ± SEM. *p < 0.05, **p < 0.01, ***p < 0.001, ****p < 0.0001.

bioRxiv preprint doi: https://doi.org/10.1101/2023.03.01.530608; this version posted March 2, 2023. The copyright holder for this preprint (which was not certified by peer review) is the author/funder, who has granted bioRxiv a license to display the preprint in perpetuity. It is made available under aCC-BY 4.0 International license.

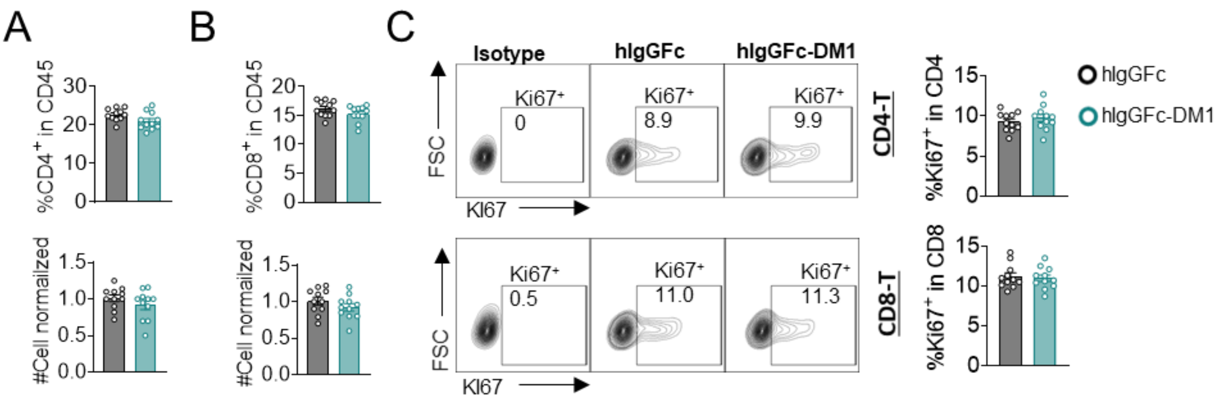

Figure 3-figure supplement 1. T-cell quantity and proliferation are not impacted by IgG-DM1 treatment.

(A) % CD4$^+$ in CD45 and normalized cell number. (B) % CD8$^+$ in CD45 and normalized cell number. (C) % Ki67$^+$ in CD4 and CD8 T cells. Mice treated with hIgGFc or hIgGFc-DM1, data combined from two independent experiments (n=11) and analyzed using an unpaired two-tailed Student's t test and represented as mean ± SEM. *p < 0.05, **p < 0.01, ***p < 0.001, ****p < 0.0001.

bioRxiv preprint doi: https://doi.org/10.1101/2023.03.01.530608; this version posted March 2, 2023. The copyright holder for this preprint (which was not certified by peer review) is the author/funder, who has granted bioRxiv a license to display the preprint in perpetuity. It is made available under aCC-BY 4.0 International license.

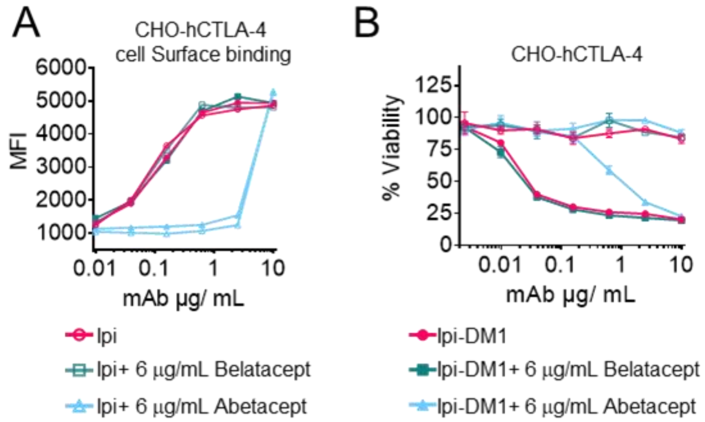

Figure 5-figure supplement 1. Mutant soluble CTLA-4-Ig does not neutralize Ipilimumab or its drug-conjugate.

(A) Detached CHO cells expressing hCTLA-4 were incubated with serial dilution of Ipi or Ipi-DM1 in the presence of given dose of Abatacept or Belatacept on ice for 30 minutes followed by FACS detection using mouse anti-human IgG AF488, mean fluorescence intensity (MFI) . (B) MTT cell viability of CHO-hCTLA-4 cells after 72 hours incubation with Ipi or Ipi-DM1 in the presence of given dose of Abatacept or Belatacept. Data is representative of two independent experiments (n=3).

bioRxiv preprint doi: https://doi.org/10.1101/2023.03.01.530608; this version posted March 2, 2023. The copyright holder for this preprint (which was not certified by peer review) is the author/funder, who has granted bioRxiv a license to display the preprint in perpetuity. It is made available under aCC-BY 4.0 International license.

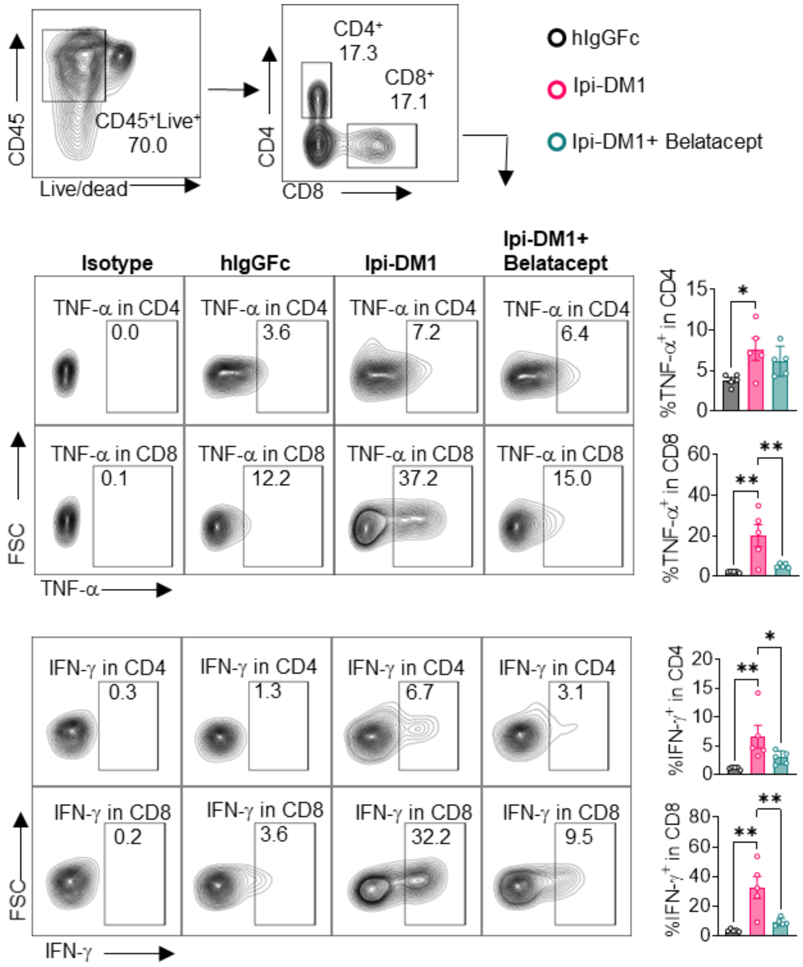

Figure 5-figure supplement 2. T-cell cytokine production.

Day 13 peripheral blood samples from treated (hIgGFc or Ipi-DM1 with/out Belatacept) mice post ACK buffer red blood lysis were cultured and stimulated in the presence of Iononmycin/PMA, and GolgiPlug for 4 hrs followed by intracellular cytokine detection (TNF-alpha, IFN-gamma) in CD4 and CD8 T cells. Data is representative of two independent experiments (n=5) analyzed by ordinary one-way ANOVA with Tukey's multiple comparisons test and represented as mean ± SEM. *$p < 0.05$, **$p < 0.01$, ***$p < 0.001$, ****$p < 0.0001$.

bioRxiv preprint doi: https://doi.org/10.1101/2023.03.01.530608; this version posted March 2, 2023. The copyright holder for this preprint (which was not certified by peer review) is the author/funder, who has granted bioRxiv a license to display the preprint in perpetuity. It is made available under aCC-BY 4.0 International license.

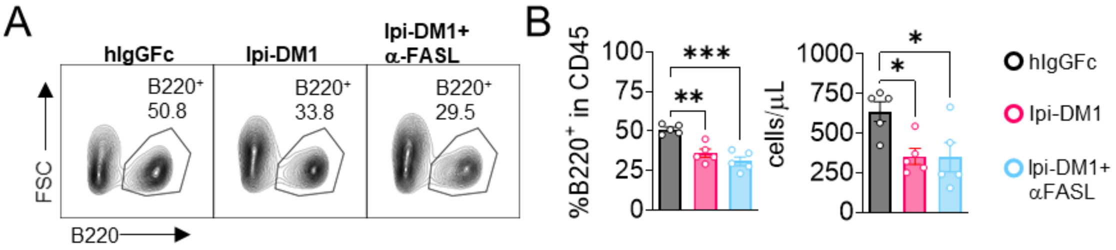

Figure 6-figure supplement 1. Anti-FASL fails to rescue B cell.

Ctla4$^{h/h}$ mice were treated intraperitoneally (i.p.) (100 μg/mouse) with hIgGFc or Ipi-DM1 with/out (100 μg/mouse of anti-FASL) every three days for a total of three doses and mice were bled on day 9. (A) FACS profile after gating on CD45 for B cells. (B) % B220$^+$ in CD45 and absolute B cell number summaries. Data is representative of one experiment (n=5). Data analyzed by ordinary one-way ANOVA with Tukey's multiple comparisons test and represented as mean ± SEM. $*p < 0.05$, $**p < 0.01$, $***p < 0.001$, $****p < 0.0001$.
